# Molecular and epidemiological characterization of human adenoviruses infection among children with acute diarrhea in Shandong Province, China

**DOI:** 10.1186/s12985-021-01666-1

**Published:** 2021-09-27

**Authors:** Deyu Huang, Zheng Wang, Guanyou Zhang, Lintao Sai

**Affiliations:** 1 Department of Infectious Diseases, Affiliated Hospital of Qingdao University, Jiangsu Road 16, Qingdao, 266000 Shandong China; 2 Department of Infectious Diseases, Qilu Hospital, Cheeloo College of Medicine, Shandong University, Wenhua Xi Road 107, Jinan, 250012 Shandong China

**Keywords:** Acute diarrhea, Children, Human adenovirus, Infection, Epidemiology

## Abstract

**Background:**

Human adenovirus (HAdV) had been recognized as one of the most common enteric viruses associated with acute diarrhea in children. The present study was carried out to demonstrate the molecular and epidemiological characterization of HAdV Infections among children in Shandong province in China between July 2017 and June 2018.

**Methods:**

Fecal specimens were collected from children under 5 years old with acute diarrhea. DNA was extracted from the stool specimens and adenovirus DNA was detected by PCR amplification with specific primers. The amplification products were subjected to electrophoresis and visualized on a UV transilluminator. All positive RT-PCR amplification products were sequenced and the obtained sequences analyzed by MEGA (version 7.0). Demographic information and clinical manifestation data were also analyzed.

**Results:**

In total, 656 fecal specimens were collected and the overall positive rate of HAdV was 7.47%. HAdV infections were detected in all age groups, in which children aged 13–24 months presented the highest positive rate. Seasonal pattern could be observed with a peak in December, January and February. Diarrhea, vomiting, dehydration and fever were the main clinical manifestations, in which vomiting was the most common accompanied symptom. By phylogenetic analysis, four species (A, B, C, and F) were detected and seven different serotypes were identified. HAdV-41 (48.98%, 24/49) was the most common serotype followed by HAdV-3 (18.37%, 9/49), HAdV-31 (14.29%, 7/49), HAdV-7 (8.16%, 4/49), HAdV-40 (4.08%, 2/49), HAdV-1 (4.08%, 2/49) and HAdV-2 (2.04%, 1/49).

**Conclusion:**

This study indicated that HAdV infection was an important cause of acute diarrhea among children under 5 years old in Shandong province. The results will contribute to (a) increase understanding of the role of HAdV in diarrheal children and enhance identification of the predominant diarrhea pathogen for diagnosis; (b) avoid abuse of antibiotics; (c) monitor the change of prevalent HAdV serotypes and promote vaccine development and vaccination.

## Background

Acute diarrhea is a major cause of morbidity and mortality among young children worldwide. More than 50% of the acute diarrhea has been confirmed to be caused by viral pathogens [1]. Like group A rotavirus, norovirus and human astrovirus, human adenovirus has been recognized as one of the most important causative agents responsible for childhood diarrhea [2–4].

HAdV as a member of the genus Mastadenovirus in the family of *Adenoviridae* is double-stranded non-enveloped DNA virus. Since it was first isolated in 1953 by Rowe et al. [5], over one hundred serotypes had been identified and grouped into seven species (HAdV A-G) based on the biological and genetic characteristics with the development of phylogenetic and bioinformatic technology [6, 7]. Different serotypes display different tissue tropisms and may cause a variety of diseases including acute gastroenteritis, respiratory illness, hemorrhagic cystitis, nephritis or meningoencephalitis [8, 9]. Severe or disseminated infections are apt to occur in immunosuppressive and immunocompromised hosts [8]. The vast majority of HAdV infections occur in children under 5 years old because of lack of immunity. HAdV F species called enteric adenovirus was confirmed to be associated with acute gastroenteritis, in which HAdV-40 and HAdV-41 were the most common serotypes accounting for 1–20% acute diarrhea [10–13]. Other non-enteric adenovirus species, such as HAdV A (types 12, 18 and 31), HAdV C (types 1, 2 and 5) and HAdV D (types 28, 29, 30, 32 and 37), were also identified to be connected with acute gastroenteritis [14, 15].

Previous epidemiological studies had reported that high ratio of HAdV associated with acute diarrhea was detected in developing countries [16–18]. Reports from several countries including Brazil, Iraq, India, Thailand and Iran had demonstrated that the prevalence of HAdV in diarrheal cases varied from 3.9 to 34.2% [14, 19–22]. China was one of the fifteen countries with a high burden of diarrhea [23] and a previous study based on a national surveillance network for patients with acute diarrhea indicated that the overall positive rate of HAdV infection was 9.27% [24]. However, the situation of HAdV infection differed among regions depending on economic development, climatic feature and geography. Higher positive rates varied from 4.37 to 28.94% were detected in northern China [25–27], while lower positive rates varied from 3.1 to 6.29% were reported in the south of China [28–30].

Recombination of is an important force driving evolution for HAdV and contributes the genetic diversity and emergence of more severe illness [31, 32]. Dynamic and continuous surveillance of the contribution of HAdV in acute gastroenteritis is important for disease prevention and control. However, limited data was available in Shandong province [33] and there was a gap in the understanding of the HAdV infections. This present study aimed to investigate the molecular and epidemiological characterization of HAdV Infections in children with acute diarrhea in Shandong province. The results will contribute to (a) increase understanding of the role of HAdV in diarrheal children and enhance identification of the predominant diarrhea pathogen for diagnosis; (b) avoid abuse of antibiotics; (c) monitor the change of prevalent HAdV serotypes and promote vaccine development and vaccination.

## Methods

### Study population

This study was carried out between July 2017 and June 2018. All enrolled patients met the following inclusion criteria: (1) they were younger than 5 years old; (2) they had no diarrhea history for at least 1 month prior to the current symptoms; (3) they underwent ≥ 3 looser than normal or watery stools within a 24-h period; (4) there was no pus or blood in the stool sample; (5) their parent or guardian consented to participate in this study. A total of 656 patients including 439 outpatients and 217 inpatients were enrolled, who visited a doctor in the clinic or were hospitalized due to acute diarrhea other than any other diseases.

They were all from two large hospitals in Shandong province: Shandong University Qilu Hospital and Affiliated Hospital of Qingdao University. Demographic information (including age, sex, place of residence and contact phone number) and clinical manifestation data (including clinical symptoms, medical history and treatment) were recorded. Informed consent was acquired from all children’s parents. This study was approved by ethics committee on scientific research of Shandong University Qilu Hospital (KYLL-2017-612).

### Samples preparation and viral DNA extraction

Fresh fecal specimen was collected from enrolled children and made into a 10% (m/v) suspension with PBS (pH 7.2). Then, the stool suspension was centrifuged at 1500*g* for 15 min. DNA was extracted from 200 μl of the supernatant using a viral nucleic acid extraction kit (TIANGEN, Beijing, China) according to the manufacturer’s instructions. The extracted DNA was eluted in 50 μl DNase-free water and stored at − 80 °C for further detection.

### Adenoviruses detection

HAdV was detected by PCR amplification using primers Ad1 (5′-TTCCCCATGGCTCAYAACAC-3′) and Ad2 (5′-CCCTGGTAKCCRATRTTGTA-3′), which were designed to target at the hexon gene [21]. The reaction mixture was comprised of buffer 5x, 1.25 mM dNTP, 25 mM MgCl2, 1U of Tag DNA polymerase and 25 pM of each primer. The cycling program consisted of an initial denaturing step of 4 min at 94 °C, followed by 35 cycles (denaturation at 94 °C for 30 s, annealing at 55 °C for 30 s and extension at 72 °C for 1 min) and a final extension at 72 °C for 10 min. The amplification products were subjected to electrophoresis on a 1.5% agarose gel containing ethidium bromide and visualized on a UV transilluminator.

### Phylogenetic analysis

All positive RT-PCR amplification products were purified and sequenced by Sangon Biotech (Shanghai) Co., Ltd. The obtained nucleotide sequences were analyzed and compared with reference sequences retrieved from GenBank database. Phylogenetic analysis was carried out based on the partial sequence of the hexon gene by MEGA (version 7.0) using the neighbor-joining method with 1000 bootstrap replicates.

Sequences of HAdV from our study were deposited in the GenBank database under the accession numbers MZ363567-MZ363615.

### Statistical analysis

Data from this study was analyzed using SPSS (version 25.0). The results were shown as proportion or the mean value with standard deviation (SD). Chi-square test, t-test and Kruskal–Wallis test were used for comparison. Statistical significance was defined as a *P* value less than 0.05.

## Results

### Detection rate

During the 1-year study period (between July 2017 and June 2018), a total of 656 stool specimens were collected from children with acute diarrhea under 5 years old in two different hospitals. HAdVs were detected in 49 (7.47%) out of the 656 stool specimens and the mean age of children positive for HAdV was 17.90 (± 9.90) months.

Among the 656 stool specimens, 302 were collected from boys and 354 were from girls. Out of the 49 HAdV-positive children, 44.90% (22/49) were male and 55.10% (27/49) were female. The HAdV detection rates were 7.28% (22/302) in boys and 7.64% (27/354) in girls, respectively. The difference of detection rate between boy and girl was not statistically significant (*P* = 0.868).

The mean age of the 656 enrolled children was 21.56 (± 7.88) months. They were divided into four age groups: group 1 (0–6 months), group 2 (7–12 months), group 3 (13–24 months) and group 4 (25–60 months). HAdV infection could be observed in all age groups. The highest HAdV-positive rate was seen in group 3 (9.70%, 26/268) and the lowest HAdV-positive rate was in group 4 (4.76%, 7/147). The positive rates of group 1 and group 2 were 5.06% (4/79) and 7.41% (12/162), respectively. However, the different positive rates among each age group were not statistically significant (*P* = 0.245).

### Monthly distribution

**Fig. 1:**
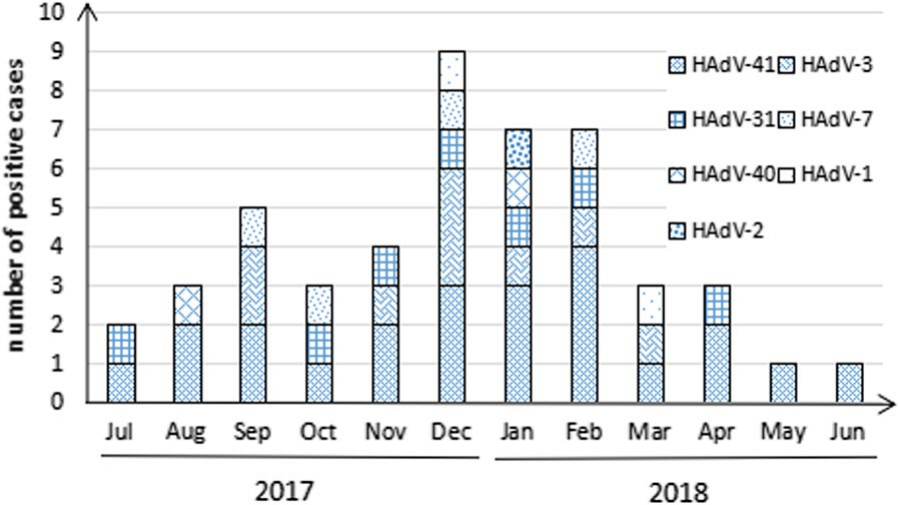
Distributions of positive cases and HAdV serotypes. HAdV infections could be observed throughout the year with a peak from December to February

HAdV infections were seen throughout the year and seasonal pattern could be observed with a peak in colder seasons (shown in Fig. 1). The higher rates of infection were found in December (18.37%, 9/49), January (14.29%, 7/49) and February (14.29%, 7/49). The lower rates of infection were observed in May (2.04%, 1/49) and June (2.04%, 1/49).

### Clinical manifestations

Diarrhea, vomiting, fever and dehydration were the common clinical manifestation for HAdV infection (shown in Table 1). The average frequency of diarrhea was 3.82 (± 1.22) episodes/day and the average duration of diarrhea was 3.02 (± 0.91) day. Vomiting occurred in 61.22% (30/49) of the HAdV-positive children. The average frequency of vomiting was 2.67 (± 0.75) episodes/day and the average duration of vomiting was 1.87 (± 0.62) days. The incidence of dehydration was 38.78% (19/49) and the mean degree was 2.10 (± 0.66) %. The incidence of fever was 44.90% (22/49) and the mean rising body temperature was 38.14 (± 0.49) °C.

**Table 1:** Comparison of clinical manifestations between HAdV-positive and HAdV-negative children

	HAdV-positive children (n = 49)	HAdV-negative children (n = 607)	*P* value
Diarrhea			
Frequency (episodes/day)	3.82 ± 1.22	4.37 ± 1.34	< 0.001
Duration (day)	3.02 ± 0.91	4.11 ± 1.12	< 0.001
Vomiting			
Incidence (%)	61.22%	74.79%	0.038
Frequency (episodes/day)	2.67 ± 0.75	2.58 ± 0.81	0.565
Duration (day)	1.87 ± 0.62	2.68 ± 0.88	< 0.001
Dehydration			
Incidence (%)	38.78%	47.94%	0.216
Degree (%)	2.10 ± 0.66	3.46 ± 0.78	< 0.001
Fever			
Incidence (%)	44.90%	65.40%	0.004
Degree (°C)	38.14 ± 0.49	38.33 ± 0.38	0.673

The main clinical manifestations of HAdV-positive children were diarrhea, vomiting, dehydration and fever. Differences of clinical manifestations between HAdV-positive children and HAdV-negative children were found by comparing frequency of diarrhea and vomiting, duration of diarrhea and vomiting, incidence of vomiting, dehydration and fever, degree of dehydration and fever. Statistical significance was defined as a *P* value less than 0.05

Obvious differences in clinical manifestation between HAdV-positive and HAdV-negative children could be seen (shown in Table 1). Frequency of diarrhea, duration of diarrhea and vomiting, incidence of vomiting and fever, degree of dehydration between the two groups were found to be statistically significant (*P* < 0.05). However, frequency of vomiting, incidence of dehydration and average body temperature of feverish children between them were not statistically significant (*P* = 0.565, *P* = 0.216 and *P* = 0.673, respectively).

### Phylogenetic analysis

**Fig. 2:**
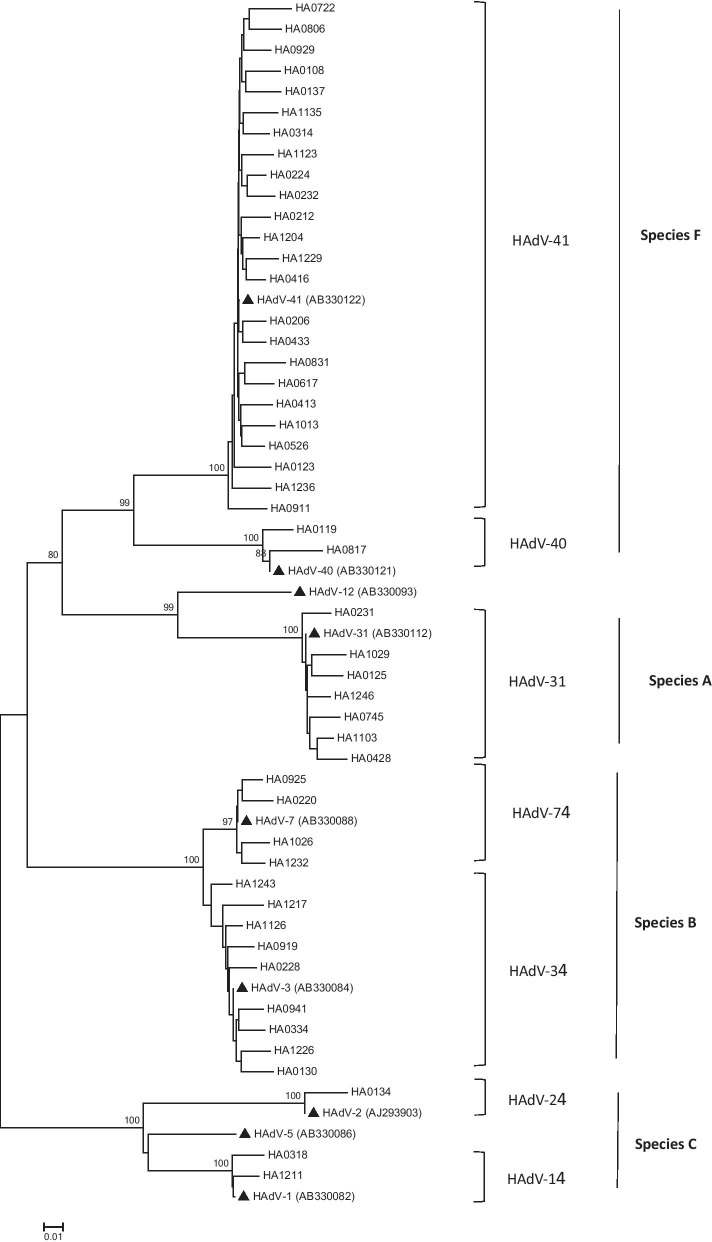
Phylogenetic tree. The phylogenetic tree was constructed based on the partial hexon nucleotide sequence (417 bp) using neighbor-joining method. The bootstrap value from 1000 replicates were shown on each branch. The referenced sequences from GenBank were marked by ▲

All the forty-nine PCR positive products were sequenced and phylogenetically analyzed. Phylogenetic tree based on the partial hexon nucleotide sequence (417 bp) was constructed to identify HAdV serotypes (shown in Fig. 2). Four species (A, B, C, and F) were detected and seven different serotypes were identified. HAdV-41 (48.98%, 24/49, species F) was the most common serotype followed by HAdV-3 (18.37%, 9/49, species B), HAdV-31 (14.29%, 7/49, species A), HAdV-7 (8.16%, 4/49, species B), HAdV-40 (4.08%, 2/49, species F) HAdV-1 (4.08%, 2/49, species C) and HAdV-2 (2.04%, 1/49, species C).

The twenty-four HAdV-41 strains detected in this study were highly similar to each other with 97.8–99.8% nucleotide sequence identity, which did not branch out as two distinct clusters. Nine samples were positive for HAdV-3 and their nucleotide sequences were closely related to the reference strain (AB330084) with 96.58–98.55% nucleotide sequence identity. Seven HAdV-31 strains were closely related to the reference strain (AB330112) with 97.43–99.13% nucleotide sequence identity. Four HAdV-7 strains were more closely related to the reference strain (AB330088) with 97.75–99.42% nucleotide sequence identity. Two HAdV-40 strains were closely related to the reference strain (AB330121) with 98.55–99.12% nucleotide sequence identity. Two HAdV-1 strains were related to the reference strain (AB330082) with 98.15–99.37% nucleotide sequence identity. One HAdV-2 strains were closely related to the reference strain (AJ293903) with 97.85% nucleotide sequence identity.

## Discussion

Diarrhea is still the second most common cause of death in children under 5 years old [16, 23]. Enteric viruses, including rotavirus, norovirus, human adenovirus and human astrovirus, were the major causative pathogens causing childhood diarrhea [34–37]. This study aimed to investigate the molecular and epidemiological characterization of HAdV infection among children with acute diarrhea in Shandong province, China. The results could lead to better understanding, preventing and controlling of HAdV infection.

The overall HAdV infection rate detected in this study was 7.47%, which was similar to those reports from some counties (Thailand, 7.2%; Korea, 5.5% and Japan, 4.8%-7.9%) [21, 38–40] and some cities in China (Beijing, 10.9%; Shanghai, 7.1% and Chongqing, 6.29%) [25, 28, 41]. However, the rate was obviously lower than that from other regions in China (Hebei province, 28.94% and Tianjin 17.62%) [26, 27]. Those results indicated that HAdV was a common causative pathogen causing acute diarrhea and should continue to be concerned.

HAdV infections occurred in each age group. The lower HAdV-positive rate in children aged 0–6 months might be explained by that breastfeeding made the antibodies transmit from mother to baby to protect them against HAdV infection. The highest HAdV-positive rate was seen in children aged 13–24 months. Children in this age group had increased outdoor activities and increased desire to eat independently, but they did not know how to pay attention to hygiene. These reasons might increase the risk of infection. The lowest HAdV-positive rate was observed in group 4 (25–60 months) and the reason might be that HAdV infection occurred in early childhood produced a certain immune effect.

In this study, the peak of HAdV infections was observed in colder season from December 2017 to February 2018. However seasonal distribution of HAdV infection remains a controversial topic. In China, Lu et al. [42] reported HAdV infection peaked in the autumn and winter in Shanghai, while Ouyang et al. [27] found HAdV infection peaked in the summer in Tianjin. Peak of HAdV infection occurred in the rainy season (from May to July) in Thailand and India [21, 43], while a seasonal pattern of HAdV infection was failed to identify in Brazil [14]. These results suggested that seasonality of HAdV infection depended on multiple interactions including climate, environment, economics and human behaviors. Anyhow, HAdV infections were found throughout the year, we should be constantly alert to the risk of infection.

The main clinical manifestations of HAdV infection included diarrhea, vomiting, dehydration and fever, in which vomiting was the most common accompanied symptom. The present study analyzed the symptoms of HAdV infection by comparing with that of diarrheal children negative for HAdV in this study, who might infect with bacterium, rotavirus or norovirus. Generally, clinical symptoms in HAdV-positive children were lighter than those in HAdV-negative children. Children positive for HAdV had lower frequency of diarrhea, less duration of diarrhea and vomiting, lighter degree of dehydration and lower incidence of vomiting and fever. Although the symptoms were usually mild and self-limited, we should still pay attention to the burden of HAdV infection on children.

A total of seven serotypes including two enteric adenovirus serotypes and five non-enteric adenovirus serotypes were confirmed by phylogenetic analysis. HAdV-41 was the most prevalent serotype which was in agreement with the results from previous studies [21, 26–28]. However, another enteric adenovirus serotype HAdV-40 was rarer than HAdV-41. This change in tendency maybe explained by an antigenic drift of HAdV-41 leading to its increase at the expense of HAdV-40 [44, 45]. HAdV of species B, including HAdV-3 and HAdV-7 serotypes, was the second leading species in this study. Although HAdV-3 and HAdV-7 strains usually caused respiratory tract infections, they had been identified to cause diarrhea in children [25, 26]. HAdV-31 strains had been found in association with diarrhea in some previous reports [46–49]. In this study, HAdV-31 was another common genotype with a rate of 14.29%, which suggested its potential role in diarrheal diseases.

Some limitations should be noted in this study. First, longer surveillance was needed to better understand the molecular and epidemiological characteristics of HAdV infections. Second, no diarrhea-free control group was included in this study. The distribution of HAdV serotypes in asymptomatic infected children was not clear. Third, although fecal specimens with pus or blood were removed, it could not excluded the possibility that some diarrheal children were caused by bacteria.

## Conclusion

HAdV infection was a common cause associated with acute diarrhea in children under 5 years old in Shandong province, China. Although the clinical symptoms were lighter, the burden brought by HAdV infection to children could not be ignored and should be paid more attention. The results will contribute to (a) increase understanding of the role of HAdV in diarrheal children and enhance identification of the predominant diarrhea pathogen for diagnosis; (b) avoid abuse of antibiotics; (c) monitor the prevalent HAdV serotypes and promote vaccine development and vaccination. However, further large-scale and multi-centric studies should be conducted to document the changes of clinical feature and genetic diversity of circulating HAdV serotypes in this region for giving inputs for prevention, control, diagnostics, treatment and vaccination strategy.

## Data Availability

The datasets used and analyzed during the current study are available from the corresponding author on reasonable request.

## Abbreviations

HAdV: Human adenovirus
PCR: Polymerase chain reaction
RT-PCR: Reverse transcription-polymerase chain reaction
SD: Standard deviation

## References

[1] Anderson EJ (2010). Prevention and treatment of viral diarrhea in pediatrics. Expert Rev Anti Infect Ther.

[2] Wilhelmi I, Roman E, Sanchez-Fauquier A (2003). Viruses causing gastroenteritis. Clin Microbiol Infect.

[3] Ulrich D (2014). Global issues related to enteric viral infections. Virusdisease.

[4] Carolina G, Maria CM, Sónia CL, Claudia I, Miguel B (2016). Etiology of diarrhea in children younger than 5 years attending the Bengo General Hospital in Angola. Pediatr Infect Dis J.

[5] Rowe WP, Huebner RJ, Gilmore LK, Parrott RH, Ward TG (1953). Isolation of a cytopathogenic agent from human adenoids undergoing spontaneous degeneration in tissue culture. Proc Soc Exp Biol Med.

[6] Ferreyra LJ, Giordano MO, Martinez LC (2015). Tracking novel adenovirus in environmental and human clinical samples: no evidence of endemic human adenovirus type 58 circulation in Cordoba city, Argentina. Epidemiol Infect.

[7] Human Adenovirus Working Group. 2021. http://hadvwg.gmu.edu/.

[8] Lynch JP, Kajon AE (2016). Adenovirus: epidemiology, global spread of novel serotypes, and advances in treatment and prevention. Semin Respir Crit Care Med.

[9] Lynch JP, Fishbein M, Echavarria M (2011). Adenovirus. Semin Respir Crit Care Med.

[10] Chiba S, Nakata S, Nakamura I, Taniguchi K, Urasawa S, Fujinaga K (1983). Outbreak of infantile gastroenteritis due to type 40 adenovirus. Lancet.

[11] Dey RS, Ghosh S, Chawla-Sarkar M, Panchalingam S, Nataro JP, Sur D (2011). Circulation of a novel pattern of infections by enteric adenovirus serotype 41 among children below 5 years of age in Kolkata, India. J Clin Microbiol.

[12] Shimizu H, Phan TG, Nishimura S, Okitsu S, Maneekarn N, Ushijima H (2007). An outbreak of adenovirus serotype 41 infection in infants and children with acute gastroenteritis in Maizuru City, Japan. Infect Genet Evol.

[13] Li P, Yang L, Guo JY, Zou WW, Xu XB, Yang XX (2017). Circulation of HAdV-41 with diverse genome types and recombination in acute gastroenteritis among children in Shanghai. Sci Rep.

[14] Dieli P, Gabriela TP, Timenetsky MDCSTT, Adriana L (2018). Surveillance and molecular characterization of human adenovirus in patients with acute gastroenteritis in the era of rotavirus vaccine, Brazil, 2012–2017. J Clin Virol.

[15] Kim JS, Lee SK, Ko DH, Hyun J, Kim HS, Song W (2017). Associations of adenovirus genotypes in Korean acute gastroenteritis patients with respiratory symptoms and intussusception. Biomed Res Int.

[16] Robert EB, Simon C, Hope LJ, Joy EL, Igor R, Diego GB (2010). Global, regional, and national causes of child mortality in 2008: a systematic analysis. Lancet.

[17] Sasirekha R, Gagandeep K (2009). Viruses causing childhood diarrhoea in the developing world. Curr Opin Infect Dis.

[18] Fletcher SM, McLaws ML, Ellis JT (2013). Prevalence of gastrointestinal pathogens in developed and developing countries: systematic review and meta-analysis. J Public Health Res.

[19] Ali H, Sam A, Bertha R, Tanya L, Mark OD, Ihab H (2019). Molecular detection and epidemiological features of selected bacterial, viral, and parasitic enteropathogens in stool specimens from children with acute diarrhea in Thi-Qar Governorate, Iraq. Int J Environ Res Public Health.

[20] Arpit KS, Subrat K, Nirmal KM, Mrutyunjay S, Priyadarshi SS (2017). Multiple etiologies of infectious diarrhea and concurrent infections in a pediatric outpatient-based screening study in Odisha, India. Gut Pathog.

[21] Kattareeya K, Pattara K, Hiroshi U, Niwat M (2019). Enteric and non-enteric adenoviruses associated with acute gastroenteritis in pediatric patients in Thailand, 2011 to 2017. PLoS ONE.

[22] Seyed DMN, Fatemeh Z, Hooman K, Mohammad RA, Rajab M, Latif G (2020). Prevalence of astrovirus, adenovirus, and sapovirus infections among Iranian children with acute gastroenteritis. Gastroenterol Hepatol Bed Bench.

[23] Walker CL, Rudan I, Liu L, Nair H, Theodoratou E, Bhutta ZA (2013). Global burden of childhood pneumonia and diarrhoea. Lancet.

[24] Wang LP, Zhou SX, Wang X, Lu QB, Shi LS, Ren X (2021). Etiological, epidemiological, and clinical features of acute diarrhea in China. Nat Commun.

[25] Liu LY, Qian Y, Zhang Y, Deng J, Jia LP, Dong HJ (2014). Adenoviruses associated with acute diarrhea in children in Beijing, China. PLoS ONE.

[26] Qiu FZ, Shen XX, Li GX, Zhao L, Chen C, Duan SX (2018). Adenovirus associated with acute diarrhea: a case-control study. BMC Infect Dis.

[27] Ouyang YB, Ma H, Jin M, Wang XW, Wang JF, Xu L (2012). Etiology and epidemiology of viral diarrhea in children under the age of five hospitalized in Tianjin, China. Arch Virol.

[28] Ren ZZ, Kong YM, Wang J, Wang QQ, Huang AL, Xu HM (2013). Etiological study of enteric viruses and the genetic diversity of norovirus, sapovirus, adenovirus, and astrovirus in children with diarrhea in Chongqing, China. BMC Infect Dis.

[29] Li W, Xiang WQ, Li CX, Xu JL, Zhou DM, Shang SQ (2020). Molecular epidemiology of rotavirus A and adenovirus among children with acute diarrhea in Hangzhou, China. Gut Pathog.

[30] Lu LJ, Zhong HQ, Xu MH, Su LY, Cao LF, Jia R (2021). Molecular and epidemiological characterization of human adenovirus and classic human astrovirus in children with acute diarrhea in Shanghai, 2017–2018. BMC Infect Dis.

[31] Christopher MR, Gurdeep S, Jeong YL, Shoaleh D, James C (2013). Molecular evolution of human adenoviruses. Sci Rep.

[32] Robinson CM, Seto D, Jones MS, Dyer DW, Chodosh J (2011). Molecular evolution of human species D adenoviruses. Infect Genet Evol.

[33] Lin L, Fu ZY, Li JS, Yang H, Liu XL, Zhang WQ (2019). Analysis of surveillance results of viral diarrhea among children under 5 years of age in Shandong province from 2012 to 2017. Chin J Exp Clin Virol.

[34] Bhattacharya R, Sahoo GC, Nayak MK, Ghosh S, Dutta P, Bhattacharya MK (2006). Molecular epidemiology of human astrovirus infections in Kolkata, India. Infect Genet Evol.

[35] Joseph AL, Elizabeth TRM, James AP, Karen LK, Ramanan L (2020). Incidence and etiology of clinically-attended, antibiotic-treated diarrhea among children under five years of age in low- and middle-income countries: evidence from the Global Enteric Multicenter Study. PLoS Negl Trop Dis.

[36] Li L, Phan TG, Nguyen TA, Kim KS, Seo JK, Shimizu H (2005). Molecular epidemiology of adenovirus infection among pediatric population with diarrhea in Asia. Microbiol Immunol.

[37] Bas BOM, Lia VDH (2016). Viruses causing gastroenteritis: the known, the new and those beyond. Viruses.

[38] Lee JI, Lee GC, Chung JY, Han TH, Lee YK, Kim MS (2012). Detection and molecular characterization of adenoviruses in Korean children hospitalized with acute gastroenteritis. Microbiol Immunol.

[39] Nakanishi K, Tsugawa T, Honma S, Nakata S, Tatsumi M, Yoto Y (2009). Detection of enteric viruses in rectal swabs from children with acute gastroenteritis attending the pediatric outpatient clinics in Sapporo, Japan. J Clin Virol.

[40] Dey SK, Hoq I, Okitsu S, Hayakawa S, Ushijima H (2013). Prevalence, seasonality, and peak age of infection of enteric adenoviruses in Japan, 1995–2009. Epidemiol Infect.

[41] Lu LJ, Jia R, Zhong HQ, Xu MH, Su LY, Cao LF (2015). Molecular characterization and multiple infections of rotavirus, norovirus, sapovirus, astrovirus and adenovirus in outpatients with sporadic gastroenteritis in Shanghai, China, 2010–2011. Arch Virol.

[42] Lu LJ, Zhong HQ, Su LY, Cao LF, Xu MH, Dong NN (2017). Detection and molecular characterization of human adenovirus infections among hospitalized children with acute diarrhea in Shanghai, China, 2006–2011. Can J Infect Dis Med Microbiol.

[43] Banerjee A, De P, Manna B, Chawla-Sarkar M (2017). Molecular characterization of enteric adenovirus genotypes 40 and 41 identified in children with acute gastroenteritis in Kolkata, India during 2013–2014. J Med Virol.

[44] Jones MS, Harrach B, Ganac RD, Gozum MM, Dela Cruz WP, Riedel B (2007). New adenovirus species found in a patient presenting with gastroenteritis. J Virol.

[45] Li L, Shimizu H, Doan LT, Tung PG, Okitsu S, Nishio O (2004). Characterizations of adenovirus type 41 isolates from children with acute gastroenteritis in Japan, Vietnam, and Korea. J Clin Microbiol.

[46] Grydsuk JD, Fortsas E, Petric M, Brown M (1996). Common epitope on protein VI of enteric adenoviruses from subgenera A and F. J Gen Virol.

[47] Ghebremedhin B (2014). Human adenovirus: viral pathogen with increasing importance. Eur J Microbiol Immunol.

[48] Bordigoni P, Carret AS, Venard V, Witz F, Le Faou A (2001). Treatment of adenovirus infections in patients undergoing allogeneic hematopoietic stem cell transplantation. Clin Infect Dis.

[49] Venard V, Carret A, Corsaro D, Bordigoni P, Le Faou A (2000). Genotyping of adenoviruses isolated in an outbreak in a bone marrow transplant unit shows that diverse strains are involved. J Hosp Infect.

